# Impact of the Tobacco Heating System and Cigarette Smoking on the Oral Cavity: A Pilot Study

**DOI:** 10.3390/dj11110251

**Published:** 2023-10-27

**Authors:** Ella Sever, Elvis Božac, Ema Saltović, Sunčana Simonić-Kocijan, Martina Brumini, Irena Glažar

**Affiliations:** 1 Clinic of Dental Medicine, Clinical Hospital Center Rijeka, Krešimirova 40, 51000 Rijeka, Croatia; ema.saltovic@uniri.hr (E.S.); suncanask@fdmri.uniri.hr (S.S.-K.); 2 Department of Restorative Dentistry and Endodontics, Faculty of Dental Medicine, University of Rijeka, Krešimirova 40, 51000 Rijeka, Croatia; elvis.bozac@fdmri.uniri.hr; 3 Department of Prosthodontics, Faculty of Dental Medicine, University of Rijeka, Krešimirova 40, 51000 Rijeka, Croatia; 4 Department of Orthodontics, Faculty of Dental Medicine, University of Rijeka, 51000 Rijeka, Croatia; martina.brumini@domzdravlja-pgz.hr; 5 Department of Oral Medicine, Faculty of Dental Medicine, University of Rijeka, Krešimirova 40, 51000 Rijeka, Croatia

**Keywords:** cigarette smoking, halitosis, oral health, tobacco products

## Abstract

Cigarette smoking and the harmful chemicals released during smoking have negative effects on oral health. As a measure of harm reduction, a new alternative tobacco heating system (THS) has been developed. The aim of the study was to analyze and compare the effects of conventional cigarettes and THS on the oral mucosa, the salivary flow rate (SFR), halitosis, and the load of *Candida* spp. The study included 20 tobacco heating smokers, 20 conventional cigarette smokers, and 20 nonsmokers. The subjects completed questionnaires on medical information, smoking habits, oral lesions, and symptoms. A clinical examination and SFR test were performed on each subject, followed by an organoleptic assessment of halitosis. Mucosal swabs were collected and cult ured to identify *Candida* spp. Significant differences were found between the smoking groups in relation to halitosis (*p* < 0.001; ε2 = 0.624), intraoral findings (*p* < 0.001; ε2 = 0.507), SFR (*p* < 0.001; ε2 = 0.0331) and dry mouth for subjective complaints (*p* = 0.021; ε2 = 0.363). The SFR was significantly lower; however, halitosis, the prevalence of intraoral findings, and dry mouth were significantly higher among smokers, but there were no significant differences between THS and conventional smokers. The present study suggests that THS smoking has similar effects on oral tissues, especially the SFR and halitosis, as conventional cigarette smoking.

## 1. Introduction

Cigarettes contain a variety of harmful and potentially harmful constituents that have adverse health effects. Smoking is associated with an increased risk of developing cancer, as well as cardiovascular, respiratory, and oral diseases. As a harm reduction measure and to reduce exposure to these harmful and potentially harmful substances, alternative smoking devices such as the Tobacco Heating System (THS) have been developed [1,2]. This new THS was developed by Philip Morris International and provides a different mechanism that produces a lower temperature and avoids combustion, thus reducing the number of harmful and potentially harmful constituents and, consequently, tobacco-related diseases [3,4]. Knowledge about the health hazards of THS smoking is limited, and claims of lower risk from THS products compared with conventional cigarettes are based almost exclusively on industry-funded research. Awareness and use of THS products are steadily increasing, especially among young adults [1].

Tobacco use can affect the oral mucosa, teeth, and their supporting structures. The oral tissue may be affected by heat and mechanical irritation of cigarette smoke, but the most significant pathology results from chemical irritation and molecular interactions with harmful constituents that increase inflammation and cause potentially malignant or malignant conditions. The oral mucosa responds to local irritation via morphologic changes (e.g., keratinization, hyperkeratosis, atrophy, metaplasia). These changes can lead to different types of conditions that are associated with tobacco use (e.g., smoker’s melanosis, black hairy tongue, superficial glossitis, leukoedema, nicotine stomatitis, and premalignant and malignant lesions) [4,5,6,7].

Saliva is a complex body fluid composed of various electrolytes, peptides, glycoproteins, lipids, and water. Its functions are to protect the oral mucosa, teeth remineralization, digestion, taste sensation, and pH balance. It is the first biological fluid that comes into contact with tobacco, which is responsible for structural and functional changes in saliva in terms of reduced quantity of saliva. The main constituent of tobacco is nicotine, which can stimulate taste receptors, certain cholinergic receptors, and blood flow to the salivary glands, causing changes in salivary secretion. The mechanical, chemical, and thermal stimulation of salivary glands by cigarettes during smoking can stimulate a short-term increase in the amount of saliva. In the short term, some studies have shown an increase in salivary flow as a result of stimulation, but the long-term effect of tobacco use on the SFR remains unclear [8,9,10]. There is evidence that long-term smoking is one of the external factors that reduces saliva production and causes hyposalivation. Hyposalivation is caused by numerous factors, such as salivary gland disease, various organic diseases, treatment with chemotherapy, radiation, or can be a side effect of various medications [8]. Conventional cigarette smokers often experience a subjective feeling of xerostomia and halitosis.

Halitosis or unpleasant odors from the oral cavity are strongly associated with cigarette smoking. Previous research shows that smoking is the third leading cause of oral malodor, after periodontal disease and food stagnation. Smoking causes changes in the commensal population of normal flora in the oral cavity, leading to an increase in pathogenic microbes and the formation of biofilms on oral cells. Moreover, a number of studies have reported that smoking increases the probability of developing extensive disease and causes significant disruption in the oral environment. Another way in which conventional cigarette smoking can lead to bad breath is via the exhalation of substances from tobacco combustion, which are absorbed into the blood through the oral mucosa and then exhaled due to blood-air exchange in the lungs. In addition, smoking contributes to halitosis by causing hyposalivation [11]. 

The salivary microbiome, including *Candida* spp., is affected by many variables, including smoking. Several studies have shown a higher incidence of *Candida* species among conventional cigarette smokers. Higher presence and overgrowth of *Candida* species can lead to oral candidiasis, which can manifest itself as erythema, white plaque, thrush, coated tongue, angular cheilitis, redness of the tongue, or oral mucosa [12]. Although the exact mechanism is not yet known, research has shown that smoking conventional cigarettes causes local epithelial changes and alters local and systemic immunological responses by decreasing salivary immunoglobulin A and impairing neutrophil function. Another theory is that cigarette smoking decreases the SFR and, consequently, lowers salivary pH, which may favor the development of candidiasis [13].

The aim of the study was to analyze and compare the effects of conventional cigarettes and THSs on the oral mucosa, the SFR, halitosis, and the load of *Candida* spp. 

## 2. Materials and Methods

### 2.1. Study Participants and Selection Criteria

This stratified cross-sectional study included a total of 60 subjects from the Clinic of Dental Medicine, Clinical Hospital Center, Rijeka, Croatia, and the Faculty of Dental Medicine in Rijeka, Croatia. The participants were divided into three groups: THS smokers (n = 20), conventional cigarette smokers (n = 20), and nonsmokers (n = 20). 

The inclusion criteria for the smokers’ groups were an age of 18 or older, a positive smoking history, and a negative history of diseases. Participants were defined as nonsmokers if they had no history of smoking, smokers were defined as such if they had smoked a total of ≥100 cigarettes after starting smoking, and THS smokers were identified if they had smoked a total of ≥100 HEETs after starting smoking and had not smoked conventional cigarettes in the past six months. The lifetime exposure to smoking was calculated using the Brinkman index (BI) [14,15]. The inclusion criteria for the nonsmokers’ group were the same, except for a negative history of smoking.

The exclusion criteria were the use of other forms of tobacco, dual users (current, simultaneous use of conventional cigarettes and THS products), diseases affecting the salivary flow, candidal load, halitosis, and medication (topical or systemic antifungals, antibiotics or mouthwashes used in the past 30 days). In terms of systemic diseases and medication, subjects suffering from local inflammatory conditions (periodontitis, ANUG, salivary gland diseases, erythema multiforme, pemphigus, lichen planus) or systemic diseases (diabetes mellitus, malignant diseases, arterial hypertension, GERD, liver diseases, heart diseases, thyroid diseases, neurodegenerative diseases, autoimmune diseases, infectious diseases and immunocompromised or transplant patients) were excluded. Subjects who used medication from the following groups were also excluded (anticholinergics, antiparkinsonics, antiepileptics, antihistamines, antidepressants, diuretics, antihypertensives, hypnotics, muscle relaxants, narcotics, sympathomimetics, spasmolytics, anxiolytics, bronchodilators, hypoglycaemics, statins, analgesics, anti-inflammatory drugs, retinoids and individuals undergoing chemotherapy, radiotherapy and biological therapy).

### 2.2. Questionnaire and Clinical Examination 

All participants were asked to complete a questionnaire on their general health and smoking history. The questions included general information, information about smoking (number of cigarettes smoked daily, duration of smoking), information about systemic diseases, prescribed medication, and subjective complaints associated with smoking (changes in oral mucosa, dry mouth, burning sensation, bad breath). An intraoral clinical examination of the oral mucosa was performed by one of the authors (E.S.), and clinical data were collected from patients sitting in a dental chair, illuminated with professional dental light, and using a set of standardized dental instruments.

### 2.3. Salivary Flow Rate

Whole, unstimulated saliva was collected between 9.00 a.m. and 12.00 p.m. to avoid diurnal variation. Each participant was instructed not to eat, drink, brush their teeth, or smoke for 60 min before and during saliva collection. Saliva was collected using the spitting method for five minutes, and the SFR was calculated by dividing the collected salivary volume by the time used to collect saliva. During saliva collection, subjects were instructed not to speak or swallow [16]. Hyposalivation was diagnosed when the SFR was less than 1 mL per five minutes. 

### 2.4. Halitosis

Halitosis was evaluated using the organoleptic method. The assessment was performed by two authors (E.S., I.G.). Calibration was performed using an olfactory test (Sniffin’ Sticks Screening test, (Burghart Messtechnik, Wedel, Germany)), and interexaminer agreement was assessed in relation to 10 volunteers not included in the study using the Kappa test (κ = 0.90). Authors were instructed to refrain from drinking coffee, smoking, or wearing scented personal-care products prior to the examination.

When utilizing the organoleptic method, subjects were instructed to close their mouth for two minutes and breathe through their nose. While the subjects exhaled slowly through the mouth, at 5 to 10 cm from the examiner’s nose, each examiner performed the evaluation individually. The odor was graded on a scale of 0 to 5 as follows: 0: no appreciable odor; 1: barely noticeable odor; 2: slight but clearly noticeable odor; 3: moderate odor; 4: strong odor and 5: extremely foul odor [17]. 

### 2.5. Cultivation and Identification of Candida *spp.*

Mucosal swabs were obtained from the oral mucosa using sterile cotton swabs and were immediately inoculated onto Chromagar Candida (CHROMagar, Paris, France). The cultures were incubated at 37 °C for 48 h according to the manufacturer’s guidelines. The number of colonies was expressed as colony-forming units per swab (CFU/swab). In the absence of growth, plates were considered negative after this period and discarded. Cultures of yeast colonies were quantified according to the following scale: no colonies, 1–9 colonies, 10–24 colonies, 25–100 colonies, >100 colonies, and confluent growth, according to Olsen [18]. 

Oral yeast colonization was defined as the presence of yeast colonies in the oral cavity, whereas oral candidiasis was defined as the presence of *Candida* spp. in the oral cavity along with oral signs and symptoms (e.g., coated tongue, dry mouth, denture, redness of the tongue or oral mucosa, glossalgia, taste disorder, angular cheilitis, ulceration) [19].

### 2.6. Statistical Analysis 

The normality of the distribution of continuous data were checked using the Shapiro–Wilk test. Since the data did not have a normal distribution, non-parametric analysis was performed (Kruskall-Wallis and Mann–Whitney tests with Bonferroni correction for multiple comparisons). Since >20% of the expected cases had frequencies <5 and considering the small sample size, Fisher’s exact test for frequencies was performed to explore the association between smoking status and other collected data. The Z-test for proportions with Bonferroni correction for multiple comparisons was used post hoc. The effect size for the Chi-square and Fisher’s exact test was quantified by Phi, for the Kruskall-Wallis test by formula ε2 = H/[(n2 − 1)/(n + 1)] and for the Mann–Whitney test by formula r = Z/√N. The Cohen criteria were used for interpretation: r = 0.25–0.3 = small effect size, 0.3–0.5 = moderate, 0.5–0.7 = large and >0.7 = very large. For the interpretation of Phi, the same criteria were used, while for the ε2 squared values of r, statistical analysis was conducted by one of the authors (M.B.).

### 2.7. Ethical Considerations 

The study protocol was approved by the Ethical Committee of the Clinical Hospital Center, Rijeka (ethical approval code, 003-05/22-1/32) and the Ethical Committee of the Faculty of Dental Medicine, University of Rijeka (ethical approval code 602-03/22-1/75). The ethical guidelines of the Declaration of Helsinki were followed. All participants provided their informed consent before being enrolled in the study.

## 3. Results

### 3.1. Participants and Demographic Data

**Figure 1:**
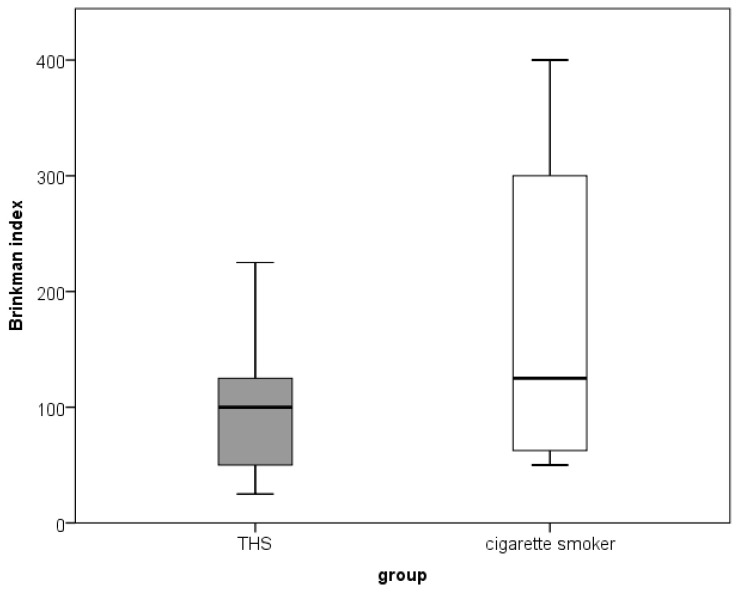
Comparison of the Brinkman smoking index between THS smokers and conventional cigarette smokers.

The demographic data of the participants are shown in Table 1. Participants were 20–56 years old (median 29; interquartile range 24–41), and 85% were female. The study included three groups of equal size (n = 20), and no statistically significant differences were found between the groups in terms of age (*p* = 0.632), sex (*p* = 1.0), education (*p* = 0.055) and lifetime exposure to smoking (the BI). The BI was lower among the THS group than among conventional cigarette smokers but was not statistically significant (Figure 1).

### 3.2. Self-Reported Oral Lesions and Symptoms

The self-reported oral lesions and symptoms are shown in Table 2. There were significant differences in the subjective, self-reported symptoms between the groups (*p* = 0.021). Participants in the conventional smokers’ group and the THS smokers’ group reported a dry mouth most frequently. Dry mouth presented with moderate side effects, and it was more prevalent among conventional cigarette smokers than nonsmokers. The THS group did not differ from the other two groups. There was no significant difference between self-reported halitosis (bad breath) and burning sensations among the groups (*p* = 0.062).

### 3.3. Halitosis, Oral Lesions, SFR and Oral Candidiasis

Significant differences between the smoking status groups were observed in the intraoral findings, but only for halitosis among the clinical findings. Halitosis presented a large effect size. The prevalence of intraoral findings and halitosis was similar in the conventional cigarette smokers and THS groups, while these were not present in nonsmokers. None of the nonsmokers had halitosis, whereas this was equally present in both smoking groups (Table 2).

No differences were observed between groups as regards the presence of oral lesions. The most common lesions in all groups were atrophy, inflammation, erosion, mortification, or coated tongue/lingua villosa. The prevalence of oral lesions detected during the participants’ clinical examination and their corresponding smoking status are shown in Table 3.

**Figure 2:**
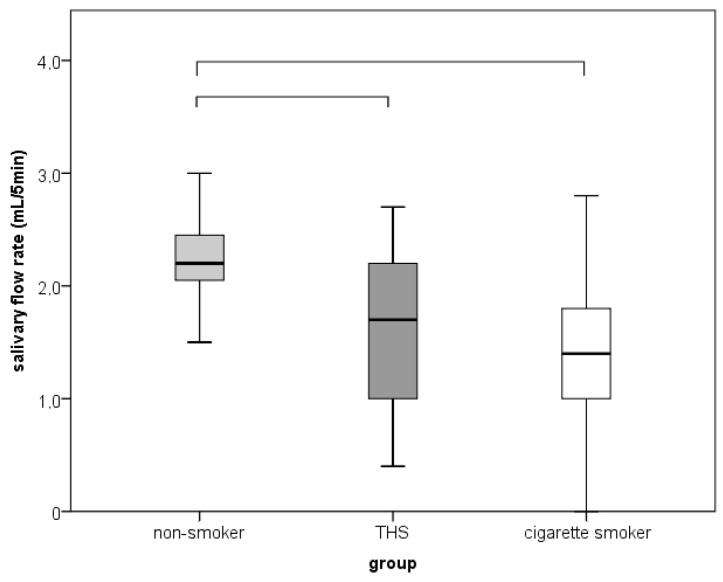
Comparison of the salivary flow rate between nonsmokers, THS smokers, and conventional cigarette smokers.

The SFR differed between groups with a large effect size (*p* < 0.001; ε2 = 0.331) and was significantly lower in the THS and conventional cigarette smokers’ groups than the nonsmokers’ group, with a larger effect among conventional cigarette smokers than THS smokers (r = 0.690 and 0.445; *p* ≤ 0.005). There were no significant differences between THS smokers and conventional cigarette smokers (Figure 2).

The oral candidiasis and burning sensation were not analyzed since these were only observed in one or two patients. Colonization with oral yeast was established in one conventional cigarette smoker (*Candida glabrata*, 500 colonies), and a burning sensation was reported by one conventional cigarette smoker and one THS smoker as an occasional and spontaneous burning sensation on the tongue with moderate (4.5 on the visual analog scale, VAS) and mild (2 on VAS) pain intensity, respectively.

## 4. Discussion

In this small, stratified, cross-sectional study, the effects of the THS on the oral mucosa, SFR, halitosis, and oral Candida load were investigated and compared with those of conventional cigarette smoking. According to the literature, THS is a relatively new tobacco product, with reduced levels of potentially harmful and harmful constituents and, consequently, a lower risk of developing tobacco-related diseases when compared with conventional cigarettes. To date, there are only a limited number of studies corresponding to the oral health effects of this alternative THS, and future, independent, in vivo investigations are needed [2,4].

The global prevalence of use of conventional cigarettes and THS products is currently higher among men. Awareness and use of THS products have been steadily increasing, particularly among young adult smokers. A large cross-sectional study of 7714 subjects in Japan found that 5.0% of men and 2.2% of women used THS products. The authors concluded that men and younger people in their 20s and 30s were more likely to smoke THS products compared with women and older age groups. Our results partially support this conclusion, including the fact that mostly younger female subjects were more likely to use THS products [20]. 

Cigarette smoking has detrimental effects on oral health and has been associated with an increased risk of oral diseases, such as premalignant and malignant lesions. According to the literature, inhaled constituents induce inflammatory and structural changes in the oral epithelium and cause histopathological alternations [4]. A large, cross-sectional study showed that smoking is a major risk factor for the development of oral lesions, not only for premalignant and malignant lesions but also for many other oral changes such as leukoedema, lichen planus, smoker’s palate, and smoker’s melanosis [21]. The results of our study are partially supportive. Although the prevalence of our intraoral findings was significant between smoking groups (THS smokers and conventional cigarette smokers), there were no changes directly related to smoking. On the other hand, the in vitro study on buccal and gingival cultures showed only minor histopathological changes in the THS group. The morphologic changes in this study by Zanetti et al. [22] were more similar to those of air-exposed controls than those of conventional cigarette smoke. In our study, oral lesions were more like those found in conventional cigarette smokers. However, other studies have included more detailed analysis (e.g., pathohistological examination, cytologic examination) of the oral mucosa and comprised subjects with more experience of smoking, whereas our results were limited to clinical examinations and included younger patients with a shorter smoking experience [22,23].

Cigarette smoke can affect the quantity and quality of saliva, and there are conflicting data on the effect of long-term smoking on the SFR. While some studies have shown an increase in the SFR in the short term, the long-term effects are still unclear [24,25,26]. In a study by Khan et al. [25], salivary reflection and the SFR were not affected by long-term smoking. However, Rad et al. [12] found a significant difference between the SFR and the self-reported symptoms of dry mouth between smokers and nonsmokers, indicating that long-term tobacco use significantly reduces the SFR. Although our results are similar to research by Rad et al. [12], our results also show no difference between the three groups separately. To our knowledge, our study is one of the first to investigate the effects of THS products on the SFR and xerostomia [24]. Even though our results indicate a higher SFR in the THS group by comparison with conventional cigarette smokers, the results are not statistically significant, and future clinical investigations using a larger sample are needed. 

The results of our study showed a statistically significant difference in effect size in terms of halitosis between THS smokers, conventional cigarette smokers, and nonsmokers. The results showed that THS smokers had a lower frequency and intensity of halitosis compared with conventional cigarette smokers. A comparative study of 100 smokers and 100 nonsmokers in Malaysia by Jiun et al. [27] found that conventional cigarette smokers were significantly more likely to suffer from halitosis. Similar results were found by Rad et al. [12], who identified that long-term smoking was associated with hyposalivation, which is the one factor that causes halitosis. In contrast, a study by Gavazova et al. [28] found nonsignificant but slightly higher levels of halitosis among conventional cigarette smokers (mean halitosis level in the smokers’ group—4.1 ± 0.1; mean halitosis level of the nonsmokers 3.73 ± 0.17). The presence of halitosis among THS smokers has not previously been studied, and the decrease in intensity and prevalence may be explained by a reduction in numerous toxicants in the absence of cigarette smoke and combustion [3].

Although several studies reported that cigarette smoking was associated with a higher incidence of *Candida* species and oral candidiasis, other research, including the current study, failed to find the relationship between oral Candida loads, conventional cigarette smoking, and THS [29]. Our results are comparable with those of a study of 58 smokers and 42 nonsmokers in Brazil, in which the difference between oral Candida loads among smokers and nonsmokers was not apparent [30]. In our study, the majority of patients were young adults, most of whom had smoked for less than 10 years, in contrast to another study in which patients with oral candidiasis were moderate to heavy smokers with a longer smoking history [31]. The rate of the oral Candida load may be variable, and because of these conflicting results, further studies should be conducted.

The limitations of our study are the relatively small sample size and the fact that the study included mainly younger participants with a shorter smoking experience. Moreover, the participants’ previous history of smoking conventional cigarettes was not considered, which could be an important confounding factor and could possibly alter the results.

## 5. Conclusions

The objective of this study was to evaluate and compare the effects of conventional cigarettes and THS on oral mucosa, SFR, halitosis, and the load of *Candida* spp. Comparing the groups, statistically significant differences were found in halitosis, intraoral findings, SFR, and dry mouth among subjective complaints. Although SFR was significantly lower, halitosis, prevalence of intraoral findings, and subjective feeling of dry mouth were significantly higher in smokers, with no significant differences between THS and conventional smokers. There is still a severe lack of studies in the literature on the effects of THS products on oral health. Nevertheless, this pilot study, with its limitations, seems to show that THS smoking has similar effects to conventional cigarette smoking, especially in terms of SFR and halitosis. Therefore, future studies with larger samples are needed to investigate the effects and risks of the relatively new THS products, especially due to the rapid rise in the use of these products by younger populations.

## Figures and Tables

**Table 1:** Sociodemographic and smoking data of the participants in the nonsmokers group, THS smokers, and conventional smokers group.

Sociodemographic Characteristics	N-S ^a^	THS	CC-S	^b^ (*p*)
Age				
Median	29.5	27	29	
Minimum	20	21	22	
Maximum	55	56	56	0.632
Gender				1.00
Male/n (%)	3 (15%)	3 (15%)	3 (15%)	
Female/n (%)	17 (85%)	17 (85%)	17 (85%)	
Education				
Magister degree	11 (55%)	8 (40%)	4 (20%)	
Bachelor’s degree	1 (5%)	4 (20%)	1 (5%)	
High school	8 (40%)	8 (40%)	15 (75%)	0.055
Brinkman index				
Mean (Minimum–Maximum)	0	100 (25–225)	125 (50–400)	
Number of cigarettes/per day	0	10 (5–20)	15 (5–20)	
Duration of smoking/years	0	10 (5–15)	10 (5–20)	

^a^ N-S nonsmokers, THS—Tobacco Heating System smokers, CC-S conventional cigarette—smokers. ^b^ Fisher’s exact test (*p*-value).

**Table 2:** Associations of smoking status with subjective patients’ complaints and intraoral findings.

	^c^	N-S ^a^	THS	CC-S	^b^ (*p*)	Effect ^b^
Subjective concern	0	17 ^a^	10 ^ab^	9 ^b^	8.139	0.363
	1	3 ^a^	10 ^ab^	11 ^b^	(0.021)	
Intraoral finding	0	20 ^a^	9 ^b^	11 ^b^	18.257	0.507
	1	0 ^a^	11 ^b^	9 ^b^	(<0.001)	
Bad breath	0	20	15	16	6.115	
	1	0	5	4	(0.062)	
Halitosis	0	20 ^a^	10 ^b^	10 ^b^		
	1	0 ^a^	8 ^b^	3 ^ab^	20.987	
	2	0 ^a^	2 ^a^	5 ^a^	(<0.001)	0.624
	3	0 ^a^	0 ^a^	2 ^a^		
Dry mouth	0	19 ^a^	16 ^ab^	12 ^b^	7.075	0.348
	1	1 ^a^	4 ^ab^	8 ^b^	(0.030)	
Hyposalivation	0	20	18	14	4.745	
	1	0	2	4	(0.069)	

^a^ N-S nonsmokers, THS—Tobacco Heating System smokers, CC-S conventional cigarette—smokers. ^b^ Fisher’s exact test (*p*-value) and Phi for the effect size. Values in the same row that share the same letters did not differ significantly. ^c^ 0 = no, 1 = present.; Halitosis according to Rosenberg scale (0 = odor cannot be detected, 1 = Questionable malodor, barely detectable, 2 = slight malodor, 3 = malodor is definitely detected, 4 = strong malodor, 5 = very strong malodor).

**Table 3:** Prevalence of oral lesions in relation to smoking status.

	^c^	N-S ^a^	THS	CC-S	^b^ (*p*)
Atrophy	0	20	18	19	1.921
	1	0	2	1	(0.766)
Inflammation	0	20	20	18	2.765
	1	0	0	2	(0.322)
Erosion/ulceration	0	20	17	19	3.111
	1	0	3	1	(0.310)
Morsication	0	20	17	17	3.550
	1	0	3	3	(0.228)
Coated tongue	0	20	15	18	5.728
	1	0	5	2	(0.058)

^a^ N-S nonsmokers, THS—Tobacco Heating System smokers, CC-S conventional cigarette—smokers. ^b^ Fisher’s exact test (*p*-value). ^c^ 0 = no, 1 = present.

## Data Availability

The data presented in this study are available from the corresponding author upon request. The data are not publicly available due to privacy.
